# First observations of *Ruminapaivae* Lowe, 1861 (Mollusca, Gastropoda) in the south-east of France, based on taxonomic clarifications

**DOI:** 10.3897/BDJ.11.e98627

**Published:** 2023-06-27

**Authors:** Louis Aureglia, Jean-Baptiste Guy, Magali Deschamps-Cottin, Bruno Vila

**Affiliations:** 1 Aix Marseille Univ, IRD, LPED, Marseille, France Aix Marseille Univ, IRD, LPED Marseille France

**Keywords:** *Ruminapaivae*, morphology, anatomy, eggs, French Mediterranean area

## Abstract

**Background:**

Amongst the genus *Rumina*, *R.paivae* was decribed from North Africa for the first time by Lowe in 1861 on the basis of a limited number of samples. During the 19^th^ and 20^th^ centuries, it was described several times, under different names and different ranks leading to a taxonomic imbroglio before being forgotten. In 2002, Mienis rehabilitated *R.paivae*, but Prevot et al. (2013, 2014) considered it as a large phenotype of *R.decollata* Linnaeus (1758) on the basis of genetic and anatomical studies.

**New information:**

In this study, we present morphological and anatomical comparisons and differences between two groups of shells collected in France and considered as *R.decollata*. Using seven morphological characters related to the size and one to the microscopic sculptures of the shell and two related to the eggs and the colours of the morphs, we attribute these two groups to two morphologically described species: *R.paivae* and *R.decollata*. We propose a way to easily distinguish them from each other. With regard to their distribution, morphology and genetics, we discuss their relative systematic position. Moreover, in this study, we report for the first time *R.paivae*, a given north African taxa, in the south-ast of France, in Marseille.

## Introduction

The genus *Rumina* includes land snails with an elongated shell and a truncated apex. Of the family Subulinidae, it is the only genus adapted to dry, open and anthropogenised environments with a Mediterranean distribution (Moreno-Rueda 2002). Three species are currently recognised within this genus (Bank and Gittenberger 1993, Carr 2002, Mienis 2008): the circum-Mediterranean *R.decollata* (Linnaeus 1758); the eastern Mediterranean *R.saharica* (Pallary 1901, Mienis 2008) and the north African (Algeria, Tunisia, Morocco) *R.paivae* (Lowe 1861). Recently, a fourth species, *R.iamonae*, was described from the Balearic Islands (Cardona 2017). The present study focuses on *Rumina* species observed in France.

Taxonomically, *R.decollata* was first described as *Helixdecollata* (Linnaeus 1758) and later as *Bulimusdecollatus* (Bruguière 1827) before being assigned to the genus *Rumina* Risso, 1826 (Locard 1899). This species has also been described under a different genus in the 19^th^ century as *Obeliscusdecollatus* (Beck 1837) or *Stenogyradecollata* (Locard 1899). Due to the strong conchyliological variations of size observed, different species and varieties have been described: *Bulimusdecollatus β major* (Pfeiffer 1859), Bulimusdecollatusvar.maxima (Bourguignat 1864), *Bulimuspaivae* (Lowe 1861), Ruminadecollatavar.maura (Crosse 1873), *Ruminaatlantica* (Pallary 1901) and *Ruminadecollatapaivae* (Wenz 1923. Later, large north African specimens, averaging 41.5 mm in length and 16 mm in diameter, recognised as a separate species, *Bulimuspaivae* (Lowe 1861), were rehabilitated as *Ruminapaivae* Lowe by Mienis (2002).

The diagnoses given by Lowe discriminate *R.decollata* from *R.paivae* on the basis of conchyliological characters and microscopic sculptures of the shell. Thus, *R.paivae*, with much larger and wider shells, has more pronounced microscopic sculptures at the sutures than *R.decollata* (Linnaeus 1758, Lowe 1861). In contrast, anatomically, the genitalia of *R.decollata* (Carr 2002) and *R.paivae* (Prevot et al. 2015) are similar, while the eggs have only been studied for *R.decollata* (Batts 1957). Consequently, the two species are defined as morphological species (Rau et al. 2022).

More recently, within the genus *Rumina*, Prevot et al. (2013) identified seven molecular operational taxonomic units (MOTUs) determined using mtDNA analysis (COI, Cytb, 16S, 12S). *R.decollata* forms a paraphyletic complex consisting of six MOTUs (A-F) and *R.saharica* forms a monophyletic group represented by MOTU S. According to this study, *R.paivae* does not form a monophyletic group since it is listed within several MOTUs (C, D, Ea) amongst *R.decollata*. However, from a biological point of view, it is important to note that *R.decollata* has a mixed breeding system with a high relevance of self-fertilisation that can promote genotypic and phenotypic differentiation (see Prevot et al. (2013)).

In view of this bibliography, we collected *Rumina* specimens from the Bouches-du-Rhône (south-east France) that we distinguished, according to strongly contrasting conchyliological differences, within two respective groups, L1 and L2. First, considering all specimens together, we tested whether one or two groups could really be distinguished on the basis of their morphological measurement. Second, on the basis of morphology and anatomy and in comparison with the literature, we associated the group(s) observed with taxa already described. Finally, we discussed the systematic position of the groups as taxa and investigated whether the morpho-anatomical data would allow us to define the first occurrence of a new taxa in France.

## Materials and methods

### Collected material

Sixteen living individuals and 541 adult *Rumina* shells were collected in urban areas in parks, gardens and wastelands in Aix-en-Provence, Marseille, Trets and Salon-de-Provence (Bouches-du-Rhône, France). Within these municipalities, our research was concentrated in semi-open environments, under shrubs and hedges (see Material in Taxon treatments). To compare our two groups, we selected only adult individuals with a thickened lip at the peristome (Kat 1981). Additional living individuals were collected and reared for observation.

### Measurements

**Figure 1.**
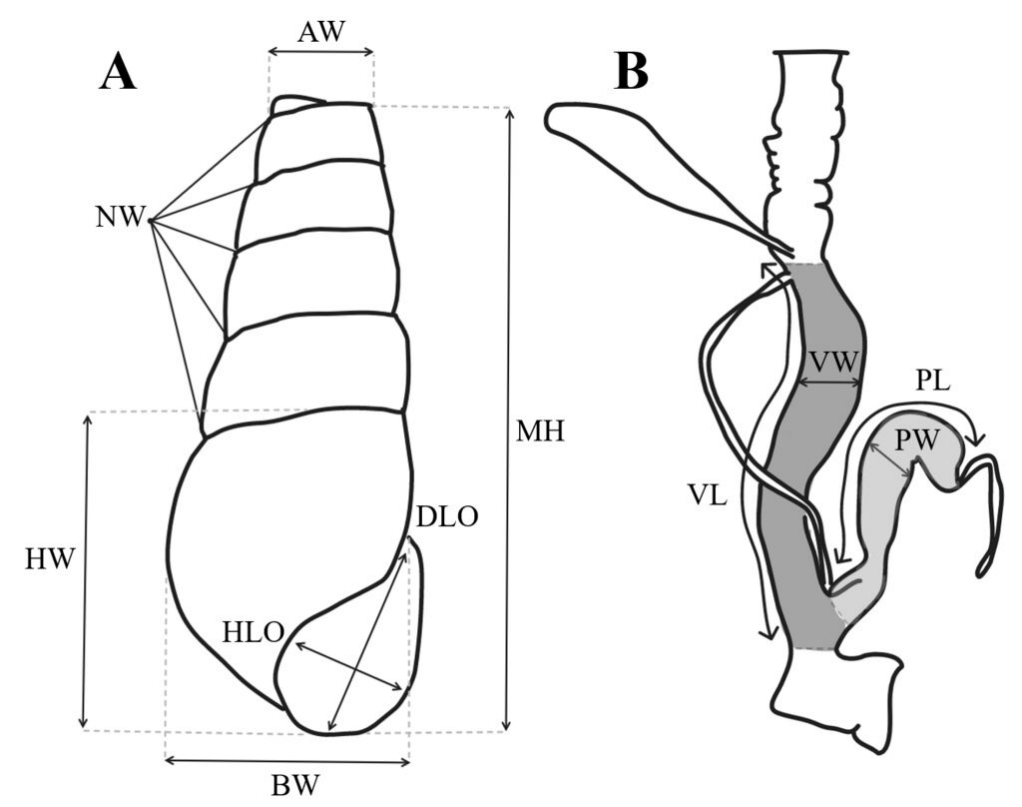
Characters studied: **A** Morphological characters measured on *Rumina* shell (adapted from Prevot et al. (2015)) and **B** anatomical characters measured on genetalia (adapted from Carr (2002)).

We measured seven characters of the shell on each of the 541 specimens collected: maximum height (MH), maximum width at the base (BW), maximum width at the apex (AW), maximum diagonal length of the aperture (DLO), maximum horizontal width of the aperture (HLO), height of the first body whorl (HW) and number of whorls (NW) (Prevot et al. 2015), using a digital caliper (DEXTER, accuracy 0.01 mm), (Fig. 1). We also observed the microscopic sculptures of the individual shells collected under the binocular magnifier (OLYMPUS SZX7).

The genitalia were studied on 16 individuals, eight individuals from group L1 and eight individuals from group L2 by measuring the length (PL) and width of the penis (PW), the length (VL) and width of the vagina (VW) (Fig. 1). Similarly, we observed the internal structures of the vaginas and penises. After dissection, genitalia were preserved in 70% alcohol to which 5% of glycerol was added.

The individuals reared having laid eggs, we carried out measurements: the diameter (ED) and the weight (EW) of eggs. The measurements were made with the digital measurement software (cellSens Entry 3.1) with a precision of 0.01 mm and the weight of the eggs with a balance (KERN EMB 100-3) with a precision of 0.001 g. Finally, when possible, we observed the body and foot colour of each living individual to associate them with a morph for each *Rumina* population. All the material (shells, genitalia and eggs) was preserved and deposited in the zoology collections of the University of Aix-Marseille.

### Groups determination and comparisons

In order to test whether one or two groups could be distinguished within the *Rumina* we collected, we carried out a cluster analysis to calculate the similarity between the individuals collected according the seven morphological criteria measured. For that, we followed the approach led by Hennig (2013). Then, the morpho-anatomical differences between the groups identified were compared for each of the measured characters with Generalised Linear Models (GLM). Post-hoc (Tukey) pairwise comparisons of estimated marginal means of fitted models were performed to compare mean Base width of shells between our two groups and *R.decollata* collected in France by Prevot et al. (2015). All tests were performed using R software v.4.1.2 (R-Core-Team 2021) and the packages emmeans (Searle et al. 1980) and ggplot2 (Wickham et al. 2016).

Finally, to put our observations into context, we compared our observations of the two groups to the morphological data of width at the base of the shells (BW) of *R.decollata* collected in France (Supplementary material S1 of Prevot et al. (2015)), corresponding to some individuals of MOTU A and Eb. In addition, we compared our results for shell base width to those reported in the diagnoses: 10 mm for *R.decollata* (Linnaeus 1758) and 16 mm for *Ruminapaivae* (Lowe 1861).

## Taxon treatments

### Rumina decollata

(Linnaeus, 1758)

58C3A2E1-CBE5-57AC-852F-CA4320C6D08F

https://doi.org/10.15468/gsw7nw

#### Description

All adult specimens in group L1 were assembled on the basis of their small size.

### Rumina paivae

(Lowe, 1861)

9279B9E4-F054-5335-A4E2-073DEE0CDE21

https://doi.org/10.15468/gsw7nw

#### Taxon discussion

All adult specimens in group L2 were assembled on the basis of their large size.

## Analysis

### Determination of groups

**Figure 2.**
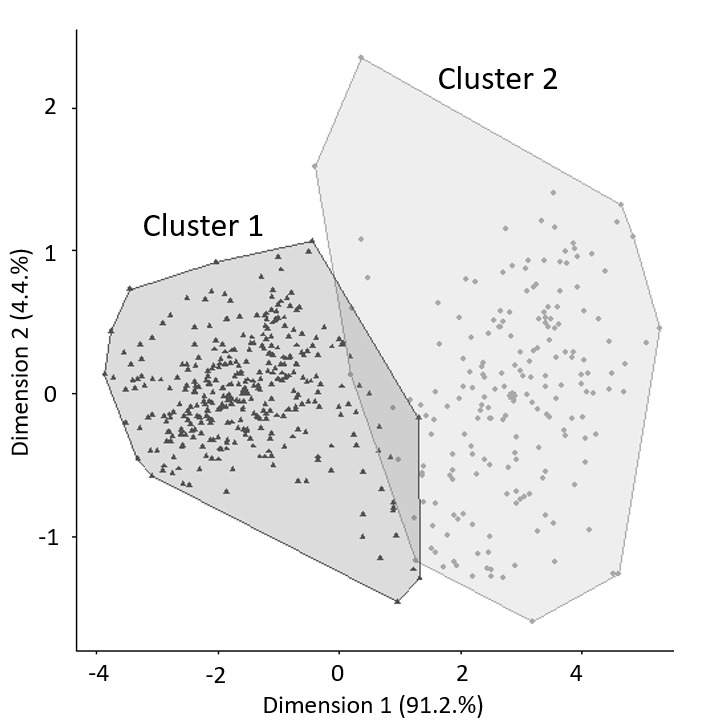
Cluster plot representing all 541 specimens of *Rumina* with dots (circles and triangles) and compared by clustering method with *k* = 2, based on seven morphological characters.

The analysis separated the 541 shells into two distinct groups on the basis of seven morphological characters. Cluster 1 contains 351 shells and cluster 2 contains 190 shells (Fig. 2). For group L1, there is a match of 93.1% with individuals from cluster 1 and, for group L2, a match of 97.4% with individuals from cluster 2. Agreement between groups 1 and 2 and the cluster solution is 0.79 using the Rand index and 0.39 using Meila’s VI. We can observe that, from the seven morphological characters, two distinct groups emerge and are statistically valid according to the cluster method with the “fpc” package (Akhanli and Hennig 2020).

### Shell measurements

**Figure 3.**
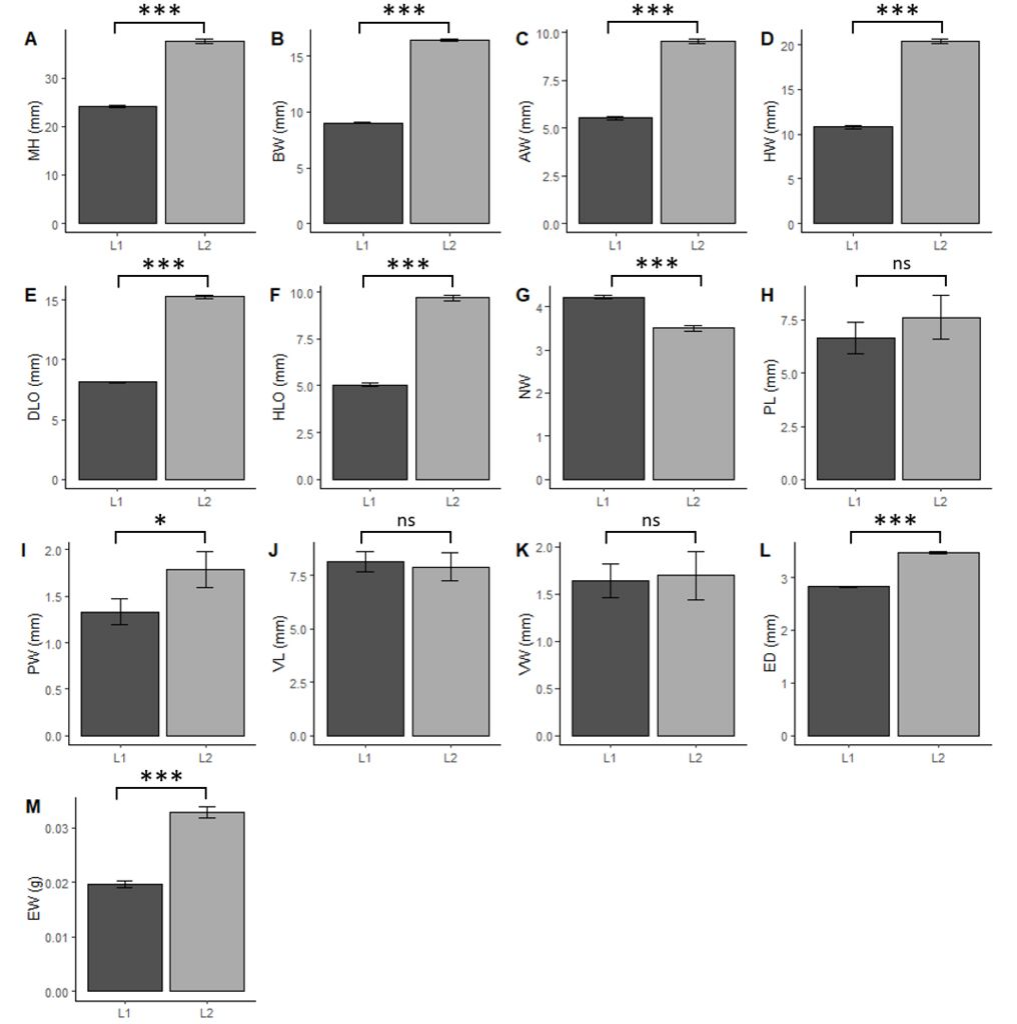
Estimated marginal means (± se) of the morpho-anatomical criteria between groups L1 and L2, based on GLM models for: **A-G** morphological values of the seven characters measured **H-I** morphological values of the eggs; **J-M** four anatomical values of the genitalia. GLM: ***, p < 0.001; *, p < 0.05; ns, p > 0.05.

**Figure 4.**
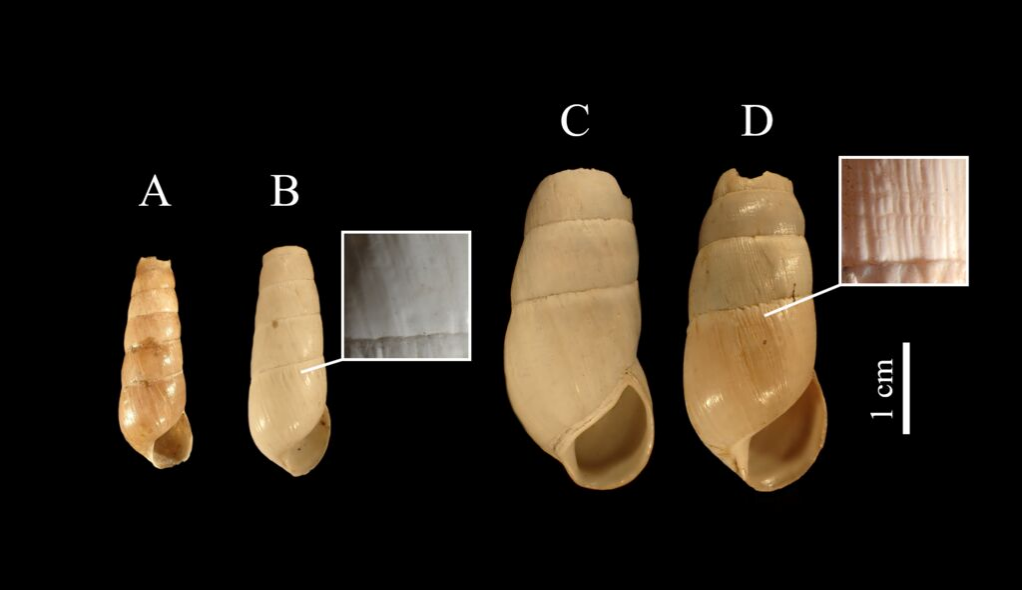
*Rumina* shells collected in Bouches-du-Rhône, views of ventral opening with details of the microscopic sculptures of the sutures: **A-B** individuals of lot L1; **C-D** individuals of lot L2; **A-D** young shells; **B-C** aged shells, (ZOO-02869 (**A**); ZOO-02860 (**B**); ZOO-02883 (**C**); ZOO-02876 (**D**)).

The morphological measurements MH, BW, AW, DLO, HLO and HW of L2 are significantly larger than those of L1 (GLM, p < 0.001, Fig. 3A-F, Table 1). As an example, the mean maximum height (MH) is 24.12 mm for the L1 group and 37.54 mm for the L2 group. Thus, the average size factor of the different measured traits is 1.8 between groups L1 and L2. Only the number of whorls (NW) varies inversely and is significantly lower in L2 than in L1 (GLM, p < 0.001, Fig. 3G). From our observations, both L1 and L2 have their own distinctive microscopic sculptures (Fig. 4). At the first suture at the base, the growth striae are more marked, tighter and hollowed for group L2, creating a dense mesh and making the shell rougher. In contrast, the growth striae for group L1 are less marked, more spaced and smoother, giving the shell a varnished aspect.

### Anatomical description

**Figure 5.**
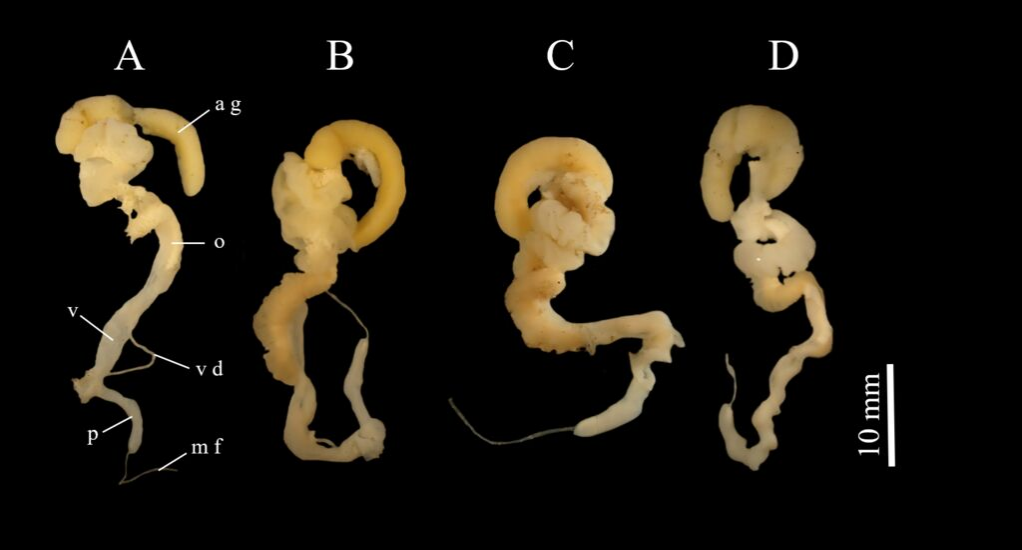
Genitalia of *Rumina* collected in the Bouches-du-Rhône. **A-B** individuals from group L1 (ZOO-02838 (**A**); ZOO-02839 (**B**)), **C-D** individuals of group L2 (ZOO-02854 (**C**); ZOO-02842 (**D**)), i.e. male flagellum (mf), penis (p), vas deferens (vd), vagina (v), oviduct (o), albumin gland (ag).

**Figure 6.**
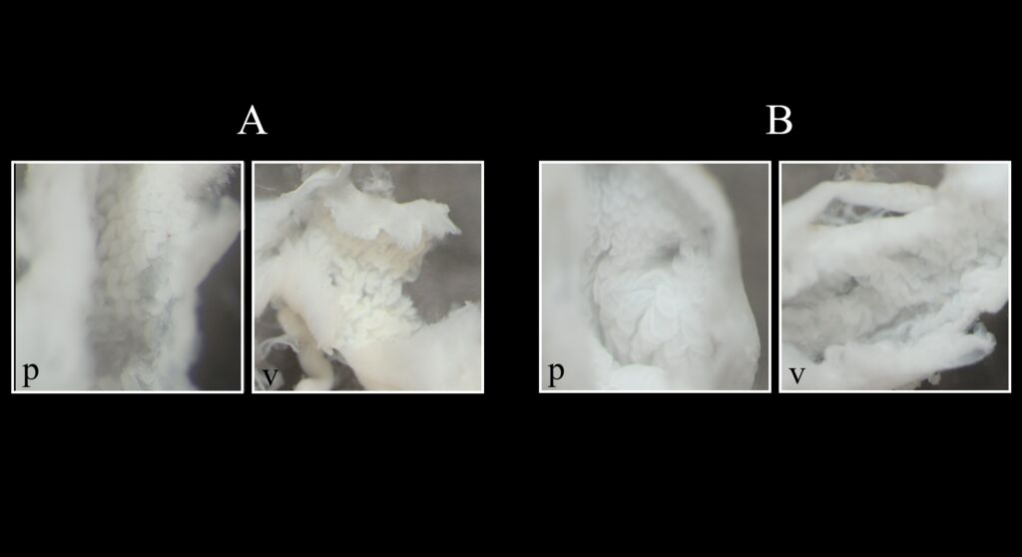
Internal structures of *Rumina* genitalia collected in the Bouches-du-Rhône with the structures of the penis (p) and the vagina (v): **A** individuals from group L1 (ZOO-02840 (p); ZOO-02846 (v)); **B** individual of group L2 (ZOO-02841).

The measurements of the genitalia, PL, PW, VL and VW, show no significant difference between the two groups (GLM, p > 0.05) (Fig. 3J-M, Table 1), except for the width of the penis (PL) which is greater for individuals from group L2 than those from group L1 (GLM, p < 0.05). The internal anatomy of the *genitalia* also revealed no differences between the two groups: we observed papillae-like structures for the penises and crenellated lamellae-like structures for the vaginas (Fig. 5, Fig. 6).

### Eggs observation

**Figure 7.**
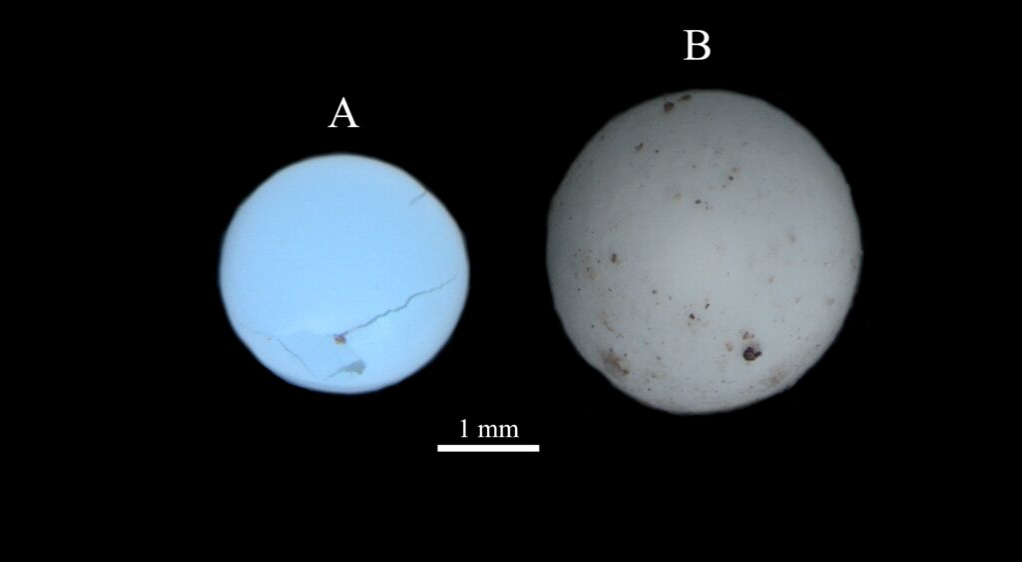
*Rumina* eggs collected in Bouches-du-Rhône. **A** from group L1 (ZOO-02843); **B** from group L2 (ZOO-02851).

Measurements of the eggs (taken 3 days after laying) show that their diameter (ED) (GLM, p < 0.001) and weight (EW) (GLM, p < 0.001) are significantly higher in L2 than in L1 (Fig. 3 H-I, Fig. 7).

### Colour morphs description

We observed in L1 two colour morphs: the light grey morph with a black medio-dorsal line (Salon-de-Provence, Trets) and the black morph (Aix-en-Provence, Marseille, Trets). In L2, only one colour morph was observed: the olive-grey morph (Marseille) (Fig. 3Table 1).

### Comparison with bibliography

**Figure 8.**
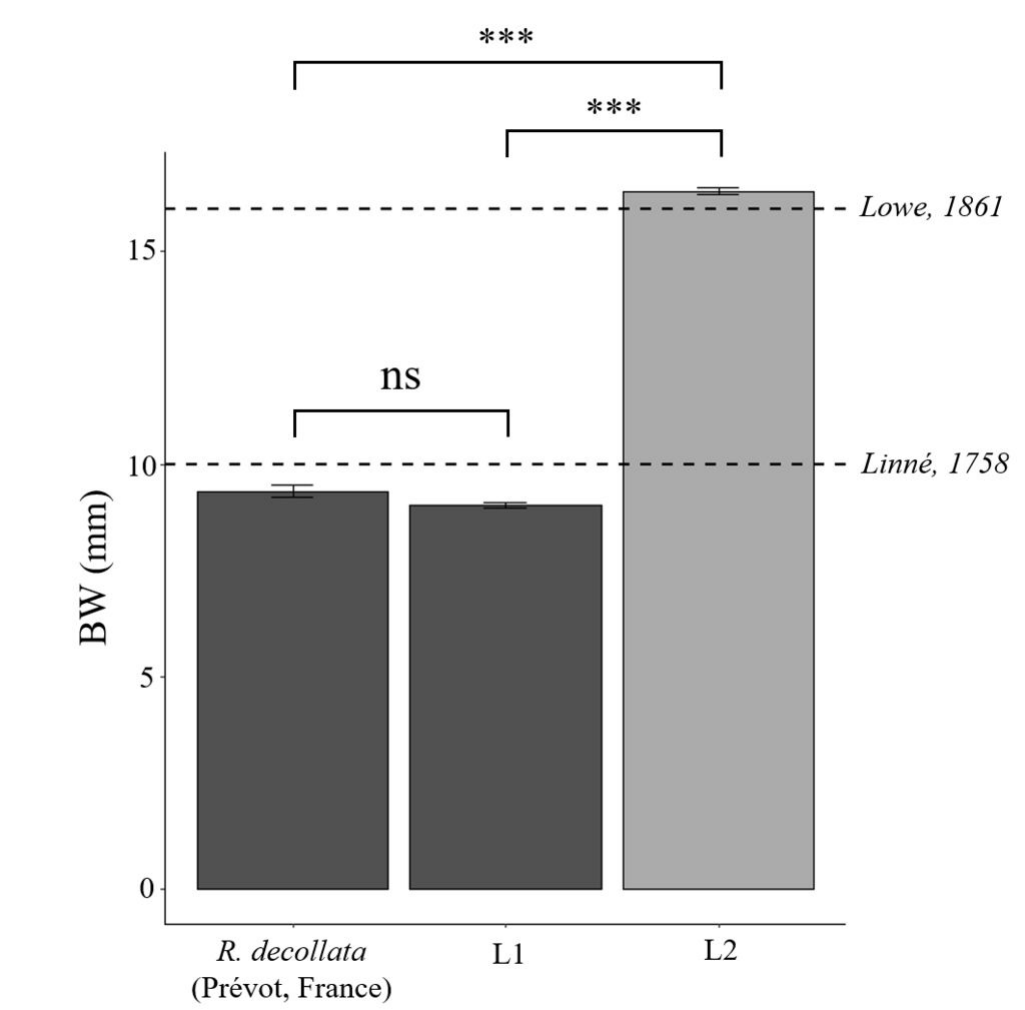
Estimated marginal means (± se) of the base width (BW), based on GLM models according to *R.decollata* collected by Prevot included in the MOTUs A and Eb (Prevot et al. 2015) and our measurements of groups L1 and L2. The horizontal dotted lines correspond to the mean BW values from the diagnoses of *R.decollata* (Linnaeus 1758) and *R.paivae* (Lowe 1861).

Finally, comparison (Fig. 8) of groups L1 and L2 for the BW trait with the Supplementary material 2 of Prevot et al. (2015) shows that group L1 is not significantly different from *R.decollata* collected in France (Tukey, p > 0.05), but that group L2 is significantly different from *R.decollata* collected in France (Tukey, p < 0.001).

## Discussion

### Characters and specific group assignment

Comparing over 500 individuals, we find highly significant differences between groups L1 and L2 for all conchyliological characters. Group L2 corresponds exactly to the measurements (BW) provided in the diagnosis of *R.paivae* (Lowe 1861), while group L1 corresponds to the diagnosis of *R.decollata* of Linnaeus (1758). Our work on a very large number of shells confirms the same measurements carried out on a limited number of individuals by Lowe (1861). Regarding the microscopic sculptures, Linnaeus describes the shells of *R.decollata* as slightly shiny with smooth whorls as well as spaced ridges, which is consistent with the characters observed for group L1. In addition, the shells of *R.paivae* are described by Lowe as opaque, imprinted with fine striae and consisting of constrictive sutures. These correspond to the characters observed for group L2.

In contrast, our measurements of the anatomical characters of the genitalia show no significant differences between groups L1 and L2, except for the width of the penis. However, this slight difference does not enable us to make it a determining character. Similarly, we do not observe any differences in the internal structures of the genitalia of our two groups, whereas these differ between *R.decollata* and *R.saharica* (Carr 2002). Our work thus fills a gap and shows that the internal anatomy of groups L1 and L2 coincides with that of *R.decollata* observed by Carr (2002).

With respect to egg diameter, those of group L1 match the diameter of *R.decollata* eggs (on average 2 mm) measured by Batts (1957). The size and weight of the eggs of group L2 being clearly greater than those of group L1, we cannot attribute group L2 to *R.decollata*, suggesting that it is another taxon. This highly discriminating character is unprecedented.

The observed body and foot colour morphs are characteristic for each of the groups: individuals from group L1 share the same morphs as French *R.decollata* from MOTUs A and Eb and those from group L2 share the same morph as *R.paivae* from MOTUs C, D and Ea (Prevot et al. 2015). No genetic differences were found between *R.paivae* and *R.decollata* by Prevot et al. (2015). Yet the morphological values of the shells of four individuals identified as *R.paivae* by Prévot and those of the L2 group match the values of the diagnosis of *R.paivae* (Lowe 1861). Thus, our results, based on shell morphology, egg characters and colour morphs, support that group L2 corresponds to *R.paivae* (Lowe 1861) and group L1 corresponds to *R.decollata* (Linnaeus 1758).

### Taxonomic rank

Like the majority of plant and animal species, the species of the genus *Rumina* have been described solely on the basis of morphological characters and correspond to the practical concept of morphological species. Recent genetic studies by Prevot et al. (2013b) have investigated the phylogenetic species concept within this genus. Involving a phylogenetic analysis of nuclear (ITS1, ITS2) and mitochondrial DNA (COI, CytB, 12S rDNA, 16S rDNA) sequences, they compared putative species in *Rumina*, inferred from the mitochondrial DNA phylogeny, with those proposed on the basis of the COI gene using five methods of comparison. These explorations generated between 7 and 17 putative phylogenetic species. Even if they argued that the data suggest at least seven species (*R.saharica*, six within *R.decollata* and rejected the species-level status of *R.paivae*), they also concluded that these methods produce a variety of different species hypotheses (Prevot et al. 2013b). Then, they explored to what extent these phylogenetic species they identified were morphologically diagnosable and to what extent they could be reconciled with the three morphological species (*R.saharica*, *R.decollata* and *R.paivae*). If *R.saharica* was already confirmed as a different species by its morphological and anatomical characters, this is not the case within the different MOTUs of *R.decollata* (*Prevot et al. 2014*).

The present study does not aim to discuss the notion of species within the genus *Rumina* since we do not have sufficient data (whether morphological, anatomical or genetic), but also and above all because the question of the notion of species is variable and difficult. The complementary elements that we can bring to the new data presented here (morphology and anatomy) concern observations relating to the biology of the species that may have repercussions on the notion of species. During our sampling, the two taxa were not found in sympatry on a local scale. This implies that the populations seem to be independent and autonomous. The absence of sympatry of the two taxa also leads us to rule out the existence of a large phenotype. However, at the same time, within the genus *Rumina*, there is the case of frequent self-fertilisation that can promote genotypic and phenotypic differentiation by fixation of alternative alleles at various nuclear gene loci (Prevot et al. 2013b).

In view of the uncertainties related to the division of *R.decollata* into MOTUs (Prevot et al. 2013a) and in the absence of proper morphological characters to distinguish them (Prevot et al. 2015), we propose to remain pragmatic and to keep the species of *Rumina* on the basis of the concept of the morphological species and to continue to use the three taxa *R.saharica*, *R.decollata* and *R.paivae*, especially when working on shells. When it is possible to identify living individuals, we also consider it important to note the colour of the morph of the body in order to relate them to the phylogenetic lineages proposed by Prevot et al. (2015).

We observe that the two groups identified for the first time in France, in Marseille (in separate localities), are easily distinguished on the basis of morphological characters and micro-sculptures of the shells. As a result, in the context of this study, we relate the two statistically identified groups to the two species already described under the names *R.decollata* Linnaeus, 1758 and *R.paivae* Lowe, 1860.

### The question of the geographical distribution of R.paivae

To date, no occurrence of *R.paivae* has been reported in France nor in Europe. Knowing that the distribution area of this species is currently north African (Algeria, Morocco, Tunisia), we report here the first occurrence of *R.paivae* in the south-east of France, in Marseille.

We can formulate two hypotheses to explain the presence of this species in Marseille:

(1) *R.paivae*, of north African origin, may be a locally introduced species due to the numerous links and exchanges between these two areas. The presence of self-fertilisation within this genus could have favoured its establishment and the maintenance of populations from a small number of individuals. We know that *R.decollata* has been accidentally introduced in the United States, Argentina, Brazil, Uruguay, South Africa, China and Japan (Prevot et al. 2014). It has also been intentionally introduced in California to regulate populations of *Cornuaspersum* (De Francesco and Lagiglia 2007).

(2) The distribution of *R.paivae* may be underestimated around the Mediterranean Basin. The species may be more common, but might go unrecorded because of the polymorphism attributed to the taxon. A good example is provided in botany with *Arundomicrantha* Lam. Described with a north African distribution, Hardion et al. (2012) have shown it is a circum-Mediterranean species. Based on data in the bibliography and our observations, the same could be true for *R.paivae*, whose range could possibly be extended to other territories, such as Jordan (Neubert et al. 2015) and the Balearic Islands (Mercadal et al. 1970, Vicente and Bech 1999).

### Perpectives

A morphological and genetic study, including a large number of specimens (body and shells) from North Africa and other countries around the Mediterranean where large forms exist, would undoubtedly shed new light on the taxonomic rank to be attributed to what we call in this publication *R.paivae*. Similarly, further research within the MOTUs of *R.decollata* would perhaps make it possible to identify distinctive characters, at least on living individuals.

## Supplementary Material

XML Treatment for Rumina decollata

XML Treatment for Rumina paivae

## Figures and Tables

**Table 1.** Characters studied (mean ± se) for groups L1 and L2: the seven morphological characters of the shell, the four characters of the genitalia and the two morphological characters of the eggs accompanied by observations of microscopic sculpture of the shells, the internal structure of the genitalia and colour morphs with number of individuals (n).

		Groups (mean ± standard error)	
		L1	L2
Shells	n	330	211
	MH (mm)	24.12 (± 4.34)	37.54 (± 5.39)
	BW (mm)	9.04 (± 1.23)	16.42 (± 1.28)
	AW (mm)	5.50 (± 0.96)	9.53 (± 1.55)
	DLO (mm)	8.12 (± 1.40)	15.26 (± 1.59)
	HLO (mm)	5.05 (± 1.22)	9.69 (± 2.10)
	HW (mm)	10.77 (± 2.39)	20.36 (± 3.74)
	NW	4.22 (± 0.82)	3.50 (± 0.58)
Genitalia	n	8	8
	PL (mm)	6.66 (± 2.14)	7.62 (± 2.03)
	PW (mm)	1.33 (± 0.38)	1.79 (± 0.41)
	VL (mm)	8.15 (± 1.35)	7.89 (± 1.34)
	VW (mm)	1.64 (± 0.61)	1.67 (± 0.39)
Eggs	n	68	55
	ED (mm)	2.82 (± 0.13)	3.47 (± 0.17)
	EW (g)	0.019 (± 0.006)	0.033 (± 0.005)
Shell sculpture	growth streaks	little marked	very marked
Genitalia structure	penis	prominent papillae	prominent papillae
	vagina	crenellated lamellae	crenellated lamellae
Colour morph	foot	black / light brown	white
	body	black / light grey with dorsal line	olive grey
